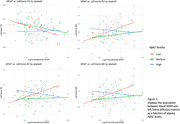# Synergistic effects of GFAP and Aβ42 on clinical outcomes: Implications for white matter microstructure and verbal memory

**DOI:** 10.1002/alz.090455

**Published:** 2025-01-09

**Authors:** Brianne M Bettcher, Dan Lopez Paniagua, Yue Wang, Brice McConnell, Christina M Coughlan, Tara C Carlisle, Ashesh A Thaker, William Lippitt, Christopher M. Filley, Victoria S Pelak, Huntington Potter, D. Adriana Solano, Jada Boyd, Nichole E Carlson

**Affiliations:** ^1^ University of Colorado Anschutz Medical Campus, Aurora, CO USA; ^2^ University of Colorado Anschutz, Aurora, CO USA

## Abstract

**Background:**

Plasma GFAP, a biomarker of astrogliosis, has previously been linked with Alzheimer’s disease clinical status, amyloid levels, and memory performance in older adults. The neuroanatomical pathways by which GFAP might impact cognitive outcomes remains unclear. We evaluated whether fornix structure, which is critically involved in AD‐associated cholinergic pathways, is associated with interactive effects of plasma GFAP and amyloid levels. We also appraised whether fornix structure mediates associations between GFAP and verbal memory.

**Method:**

In a cohort of both asymptomatic and symptomatic older adults (total n=90), we assessed GFAP, AD‐related proteins, and vascular markers in plasma, and we conducted comprehensive memory testing. Tractography‐based methods were used to assess structure of the fornix, and whole brain diffusion metrics were also ascertained to control for diffuse alterations in white matter.

**Result:**

In individuals whose blood levels of Aβ42 were in the lowest tertile, higher GFAP levels were associated with lower fractional anisotropy (FA; p=0.007), higher mean diffusivity (MD; p<0.001), higher radial diffusivity (RD; p <0.001), and higher axial diffusivity (AD; p=0.001) in the left fornix. These associations were independent of APOE gene status, plasma levels of total tau and neurofilament light, plasma vascular biomarkers, and whole brain diffusion metrics. Fornix structure partially mediated the association between higher GFAP levels and lower verbal memory performance in participants with lower plasma Aβ42.

**Conclusion:**

Results suggest that in the setting of greater amyloid deposition, higher levels of blood GFAP are associated with altered fornix microstructure. We also expanded on our prior GFAP‐verbal memory findings, demonstrating that in the subset of participants with lower blood Aβ42 levels, left fornix integrity may be a primary white matter pathway for the negative associations between GFAP and verbal memory performance. Overall, these findings suggest that GFAP may play an early, pivotal role in AD pathogenesis, and further demonstrate that the combination of blood GFAP and Aβ42 may have a particularly negative role in forniceal‐memory pathways.